# How the environment shapes our ability to navigate

**DOI:** 10.1002/ctm2.928

**Published:** 2022-06-09

**Authors:** Hugo J. Spiers, Antoine Coutrot, Michael Hornberger

**Affiliations:** ^1^ Institute of Behavioural Neuroscience, Department of Experimental Psychology, Division of Psychology and Language Sciences University College London London UK; ^2^ LIRIS–CNRS–University of Lyon Lyon France; ^3^ Norwich Medical School University of East Anglia Norwich UK

Where we grow up can define us in many ways: how we speak, what activities we do, and who we might spend our life with. We have recently found that it can also impact the ability to navigate.^1^ Growing up in a city has, on average, a negative impact on navigation skill. This insight may be important for the development of new tools to aid the diagnosis and monitoring of function in Alzheimer's disease.

## 1 THE CHALLENGE OF TESTING SPATIAL NAVIGATION

Spatial disorientation is a core early symptom of Alzheimer's disease (AD).^2^ The areas of the brain known to be important for navigation and spatial orientation are amongst the earliest to be impacted by AD pathophysiology.^2^ However, to date there have been few cognitive tests available to assess spatial navigation and orientation clinically. This is because most cognitive tests are paper and pencil based, whereas spatial navigation requires exploring an environment and making judgments about it, either in the real‐world or a virtual reality environment. Because people live in different environments with varying complexity, it is not trivial to determine whether someone has a specific problem and comparison to a cohort of other participants is rarely possible. Thus, it is useful to develop a test of navigation skill that could be taken by anyone to assess their navigation ability, potentially as part of diagnostics, but also for tracking decline and as a functional readout or end‐point assessment for AD intervention studies.

## 2 A NAVIGATION TEST EMBEDDED IN A VIDEO GAME APP

**FIGURE 1:**
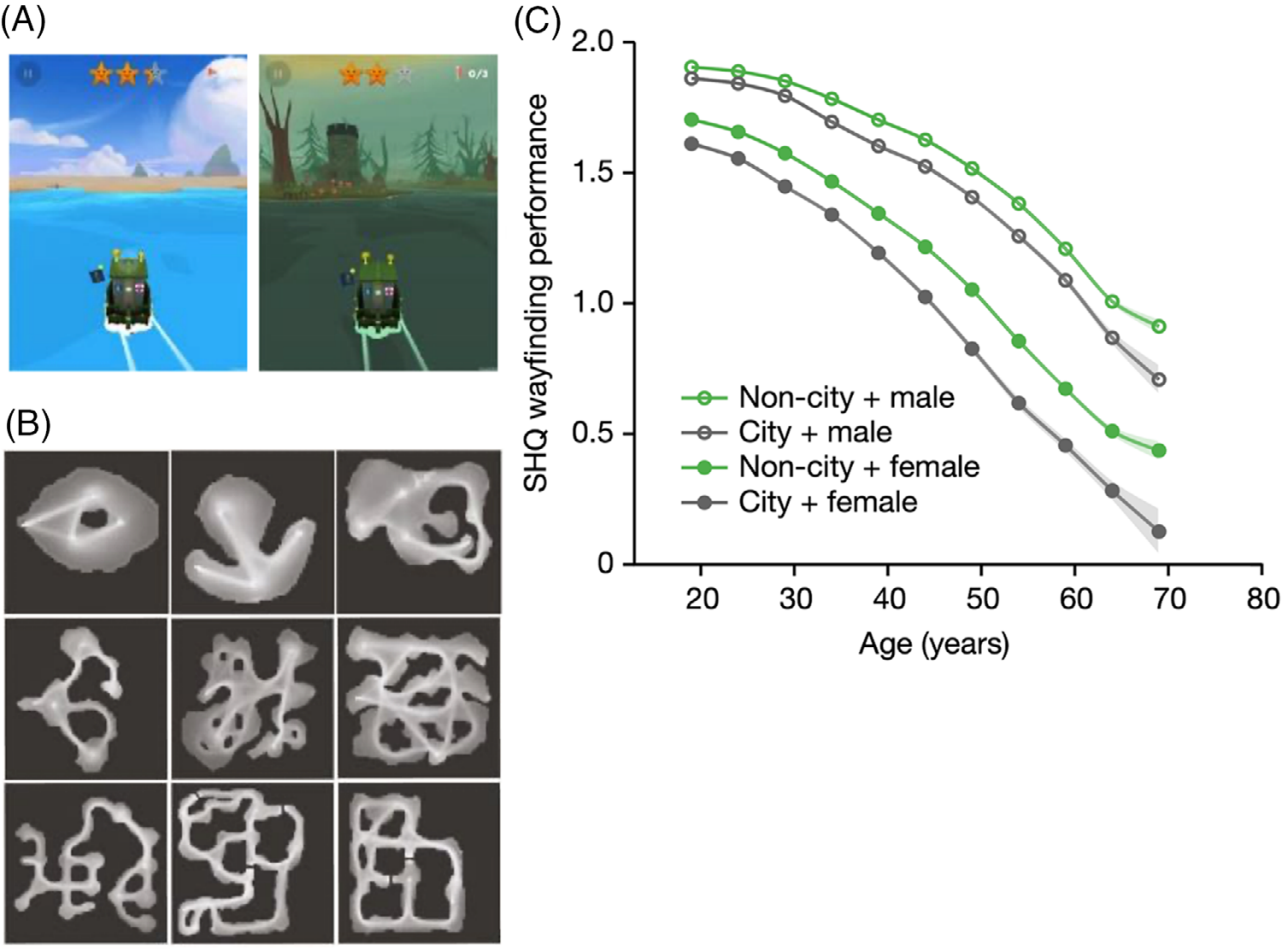
Task and evidence for the impact of cities on navigation ability. (A) Screenshots from the game Sea Hero Quest (SHQ). (B) Nine examples of trajectory heat maps out of the 75 SHQ levels. (C) Association between environment and SHQ wayfinding performance stratified by age and gender. The SHQ wayfinding performance is computed from the trajectory length and has been averaged within 5‐year windows. Error bars, standard error; centre values, mean. *Source*: Figure adapted from^1^

Creating such a test is a challenge for two reasons. First such a test requires the use of virtual environments that participants can explore and that are intuitive for people of all age ranges to operate. Second, to develop a validated test requires many participants to provide a normative sample from which to compare a patient's performance to. Because navigation ability varies with gender and age it means many participants are required to determine the normal pattern, which is costly and time‐consuming. To overcome these limitations we developed a navigation test embedded in a mobile app video game—*Sea Hero Quest* ^3^, ^4^ (see Figure 1A,B). The video game involves navigating a small boat through a range of aquatic environments in search of mystical sea creatures. Wayfinding levels present players with a map indicating check‐points to reach and the layout of the environment. After this, players tap left or right of the boat to steer to these checkpoints with their trajectory recorded and transmitted from the mobile device to a remote server for later analysis. Using this data we have revealed that navigation ability linearly declines over the life‐span, population‐level performance for a country can be predicted by its GDP and the extent of the male advantage in navigation skill can be predicted from economic disparities between countries.^3^ Navigation performance in the game is also predictive of real‐world navigation performance,^5^ shows good test‐retest reliability,^6^ and it can better distinguish those at genetic‐risk of AD than current ‘gold‐standard’ tasks.^7^

## 3 GROWING UP IN CITIES RESULTS IN WORSE NAVIGATION ABILITY

**FIGURE 2:**
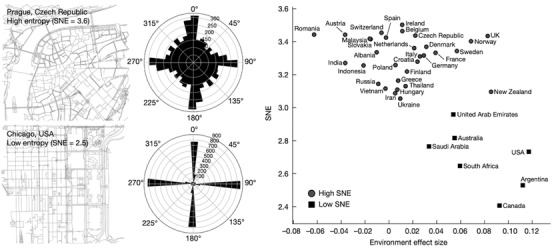
Street Network Entropy (SNE) and environment effect in 38 countries. Left, two example cities with low (Chicago, USA) and high (Prague, Czech Republic) SNE. Middle, circular plots show the distribution of the street bearings across 36 bins of 10°. Right, average SNE as a function of the environment effect size in each country. The environment effect sizes are the country slopes from a linear mixed model for wayfinding performance, with fixed effects for age, gender, and education, and random environment slopes clustered by country (*n* = 397 162 participants). Positive values indicate an advantage for participants who were raised outside cities. The average SNE is the weighted average over the 10 most populated cities of the country, weighted by their population. Squares and circles correspond to the low‐SNE and high‐SNE country groups, determined with k‐means. *Source*: Figure adapted from R^1^

Our previous results have shown that demographics (age, gender) can influence navigation performance,^3^ but it is less clear if the spatial environment people grew up in also influences their navigation behaviour. Our most recent study addressed this question directly by contrasting navigation performance of people who report growing up in a city against those who reported growing up outside cities.^1^ This is important for considering whether the environment might be important for future benchmarking of navigation ability. We found that on average people who grew up in cities were worse at navigating than those from outside cities^1^ (see Figure 1C). This difference was present in both men and women and persisted across the life‐span, but its magnitude varied across our sample of 38 countries. The effect was largest in countries such as the USA and Canada, which are famous for having many cities built on a grid‐plan. We hypothesised that the city layout can explain the negative effect of cities on navigation ability. We quantified how ‘griddy’ different cities were from the countries in our sample using a measure known as the street network entropy (SNE).^8^ SNE measures how consistently the streets in a city are oriented in the same direction. For example, Chicago with its grid layout has a very low SNE, while Prague with variably oriented streets has a much higher SNE score (Figure 2). At the country‐level we found that we could predict the negative impact of growing up in a city from the SNE average of the top ten largest cities in a country (Figure 2). Going further, we found that people who grew up in cities with griddy cities are particularly sensitive to the SNE in the different environments tested in Sea Hero Quest. Moreover, there was a slight performance advantage for people who grew up in griddy cities when the virtual game environment was very griddy. This indicates that griddy cities do not simply reduce navigation ability, but rather they shape the future navigation skill so that it is more optimized for navigating in environments similar to those people grew up in. Finally, in a follow up experiment we showed that the negative impact from cities also occurs when navigating a virtual city, and that it is the environment someone grows up in, rather than their current environment, that is important for predicting navigation ability.^1^

## 4 FUTURE OUTLOOK

These findings shed light on how the environment we grow up in impacts the development of our navigation abilities. The findings have important implications for future clinical spatial navigation test development. They show that, not only do age and gender affect spatial navigation performance, but also the environment a patient has grown up in may be important to consider. Thus, the findings will inform establishing normative thresholds for spatial navigation ability based on spatial experience, allowing a more personalized cognitive diagnostic and intervention outcome measures in the future.^7^ Future research involving agent‐based modelling to predict behaviour^9^ and trajectory analysis^10^ may pave the way to even more precise assessments.

## CONFLICT OF INTEREST

The authors declare that there is no conflict of interest that could be perceived as prejudicing the impartiality of the research reported.
